# Selection of urinary sediment miRNAs as specific biomarkers of IgA nephropathy

**DOI:** 10.1038/srep23498

**Published:** 2016-03-22

**Authors:** Zhi-Yu Duan, Guang-yan Cai, Ru Bu, Yang Lu, Kai Hou, Xiang-Mei Chen

**Affiliations:** 1 Department of Nephrology, Chinese PLA General Hospital, State Key Laboratory of Kidney Diseases, National Clinical Research Center for Kidney Diseases, Beijing, PR China

## Abstract

The miRNAs in urinary sediment are easy to obtain, which provides a new approach to searching for non-invasive biomarkers of IgA nephropathy (IgAN). Compared with normal controls (n = 3), 214 different miRNAs in the urinary sediment of IgAN (n = 9) were found by miRNA chip assay. By quantitative PCR analysis, miR-25-3p, miR-144-3p and miR-486-5p were confirmed to be significantly higher in IgAN (n = 93) than in the normal group (n = 82) or disease control (n = 40). These three miRNAs had good specificity and sensitivity for the diagnosis of IgAN by receiver operating characteristic curve analysis, in which the AUC value of miR-486-5p was the largest at 0.935. Urinary sediment miR-25-3p, miR-144-3p and miR-486-5p were demonstrated to be mainly derived from urinary erythrocytes, which were separated by CD235a magnetic beads. The increased expression of urinary erythrocyte miRNAs in IgAN patients was not associated with those in the blood erythrocytes. In addition, urinary supernatant microvesicles of miR-144-3p and miR-486-5p in the IgAN group were also significantly increased. This study showed that the miR-25-3p, miR-144-3p and miR-486-5p in urinary sediment were mainly derived from urinary erythrocytes, which could be non-invasive candidate biomarkers for IgA nephropathy.

IgA nephropathy (IgAN) is the most common primary glomerulonephritis in the world and is also one of the main causes of the end-stage renal disease (ESRD) in China^1^. The majority of IgAN is a progressive disease, and 15–40% of patients will develop into ESRD within 5–25 years after being diagnosed^2^. Although renal biopsy is the “gold standard” for diagnosing IgAN because it is an invasive test and has certain risks, it cannot be performed repeatedly whenever the illness warrants. Therefore, non-invasive biomarkers of IgAN are required for clinical practices.

The microRNAs constitute a class of endogenous non-coding RNA 20–25 nucleotides in length. In combination with the principle of complementary base pairing of the target mRNA 3′ untranslated region, miRNAs affect the level of the translation of gene expression, resulting in the target mRNA appearing to be inhibited or degraded^3^. Urinary sediment miRNAs directly originate when passing through the kidney tissue. Additionally, they have many clinical advantages, such as being non-invasive, simple and easy to obtain. Thus, they may become a new approach in the search for non-invasive biomarkers of IgAN. Some investigators have already carried out several useful attempts^4^,^5^, but these attempts still lack specific urinary sediment miRNAs for the diagnosis of IgAN.

The traditional view assumes that mature red blood cells (RBCs) are highly differentiated cells. During their maturation process, they gradually lose organelles and nucleic acids in preparation for the storage of hemoglobin^6^. Thus, according to the previous theories, erythrocytes were merely a packet filled with hemoglobin^6^,^7^. However, recent research has indicated that red blood cells contain abundant miRNAs and mature red blood cell miRNAs account for the vast majority of the entire miRNAs in the blood^8^,^9^. The role of erythrocyte-derived miRNAs in the urinary sediment in IgAN was still unknown.

At present, the study of urinary sediment miRNAs biomarkers has a common problem: the unclear cellular sources of urinary sediment miRNAs and their intervention approach. This has limited the in-depth study of specific mechanisms of urinary miRNA biomarkers. In this study, we identified specific urinary sediment miRNA biomarkers for IgA nephropathy, and further demonstrated they are mainly from urinary erythrocytes by using immunomagnetic cell separation technology.

## Results

### Candidate miRNA biomarkers of IgAN

**Figure 1:**
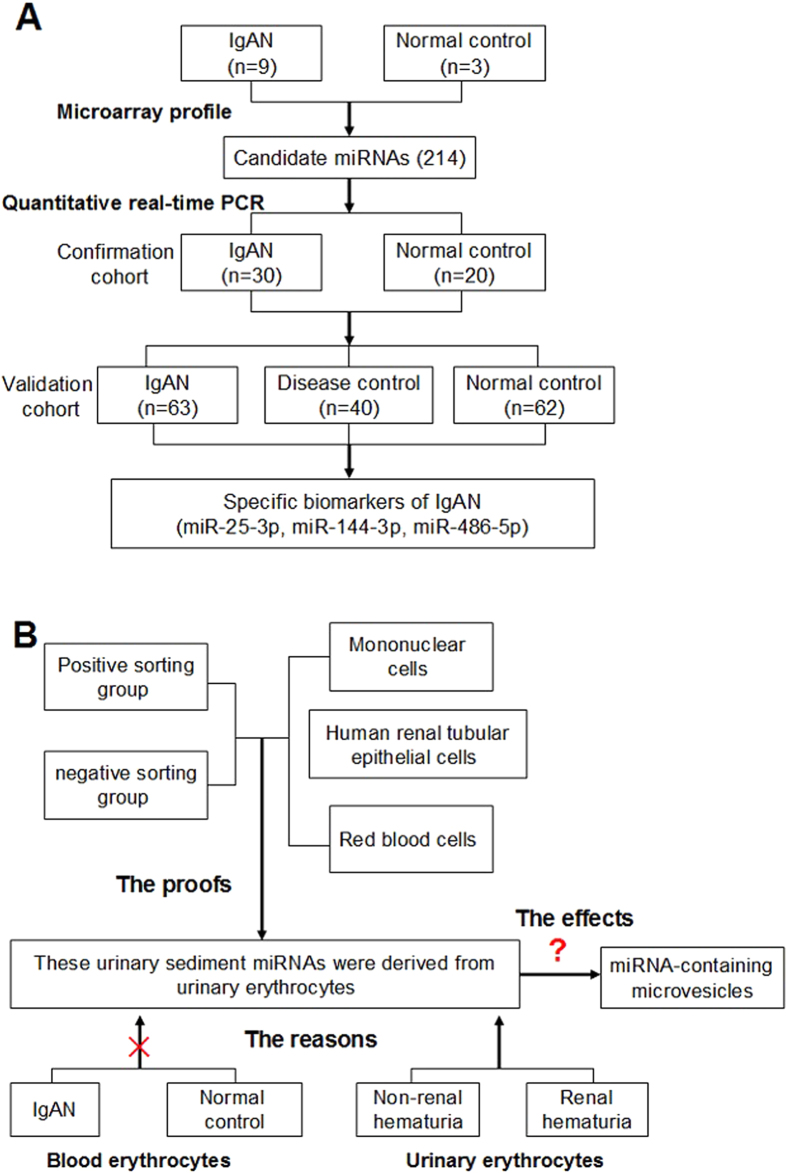
Study design. (**A**) The study of identifying biomarkers of IgAN. (**B**) The study of predicting cellular source and its possible mechanism.

**Figure 2:**
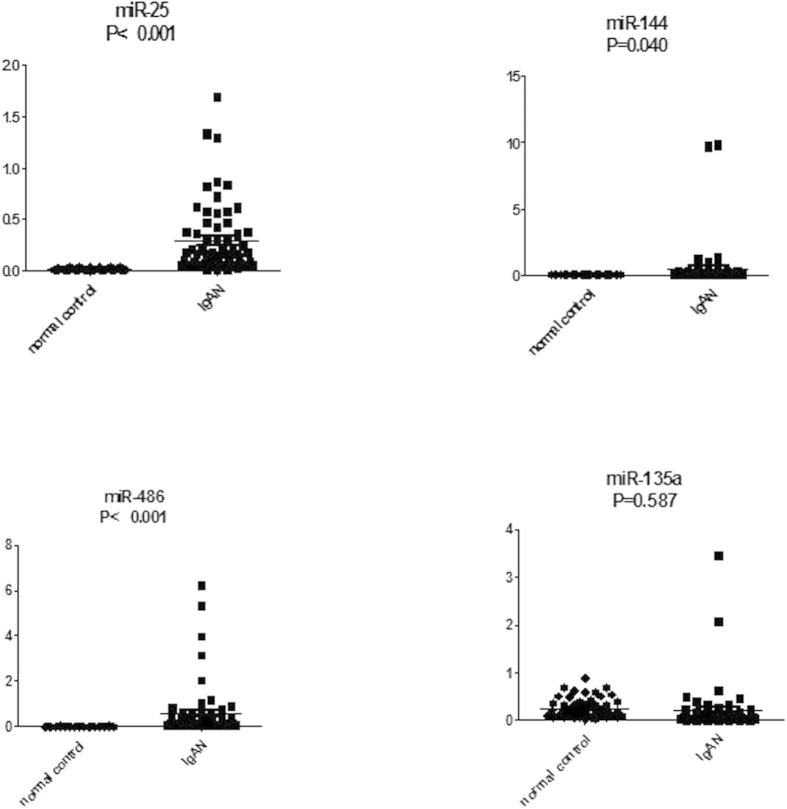
Comparison of urinary sediment miRNA expression levels between the IgAN group and normal control group in a validation cohort. Normal control, n = 62; IgAN group, n = 63. The P values were calculated by 2-tailed Student t test.

The study flow was shown in Fig. 1 (Fig. 1A,B). The urinary sediment miRNA expression profiles of IgAN were still unknown. To clarify urinary sediment miRNA expression profiles of IgAN, 12 Agilent human miRNA V19.0 chips (each chip contains 1888 types of human miRNA) were used to detect urinary sediment miRNA expression profiles in 9 IgAN patients and 3 normal controls. Compared with the normal control group, 214 miRNAs were found to differ significantly in the IgAN group, with 112 miRNAs having P-values < 0.01 (Supplementary Figure S1). We then selected 6 different miRNAs, of which three were significantly higher (hsa-miR-25-3p, hsa-miR-144-3p and hsa-miR-486-5p), and the other three were significantly lower (hsa-miR-135a-3p, hsa-miR-150-3p and hsa-miR-638) in the IgAN group. These 6 miRNAs were validated in 30 other IgAN patients and 20 normal controls (Supplementary Figure S2). The results were consistent with the trends in miRNAs chip analysis in urinary sediment. The P-values of four miRNAs (miR-25-3p, miR-144-3p-3p, miR-486-5p and miR-135a-3p) were less than 0.001. These four miRNAs were further validated in the validation cohort. The results showed that miR-25-3p (P < 0.001), miR-144-3p (P = 0.040) and miR-486-5p (P < 0.001) were significantly higher in the IgAN group than those in the normal group, while miR-135a-3p (P = 0.587) showed no significant differences between the two groups (Fig. 2). Moreover, urinary sediment miR-25-3p (P < 0.001), miR-144-3p (P = 0.046) and miR-486-5p (P = 0.010) in the IgAN group were significantly higher than those in the disease control group, and miR-135a-3p (P = 0.290) showed no significant difference (Supplementary Figure S3).

**Figure 3:**
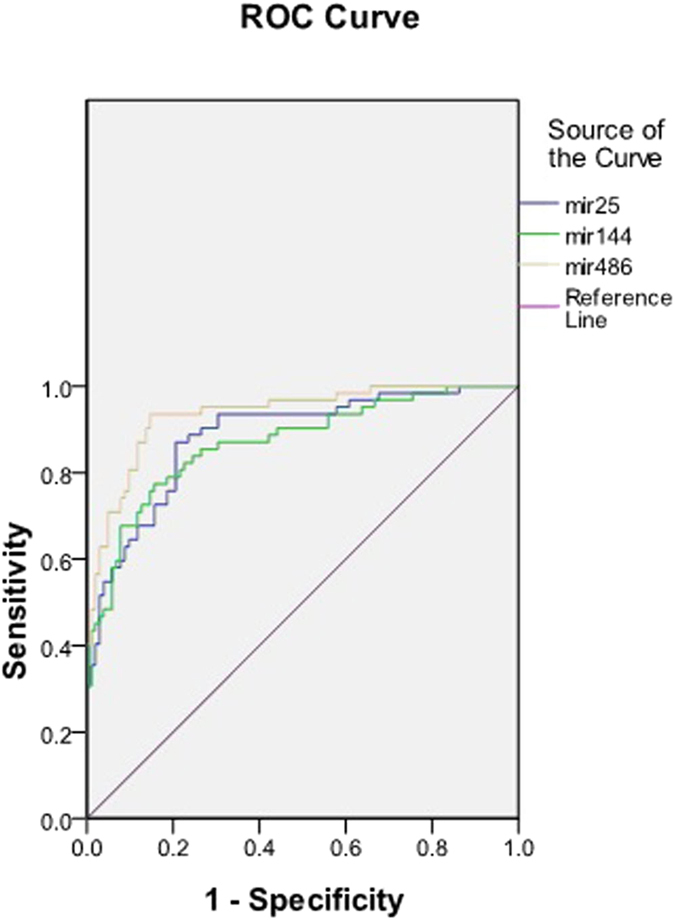
Receiver operating characteristic curve of miRNAs for diagnosing IgAN. Normal control, n = 82; disease control, n = 40; IgAN group, n = 93.

**Figure 4:**
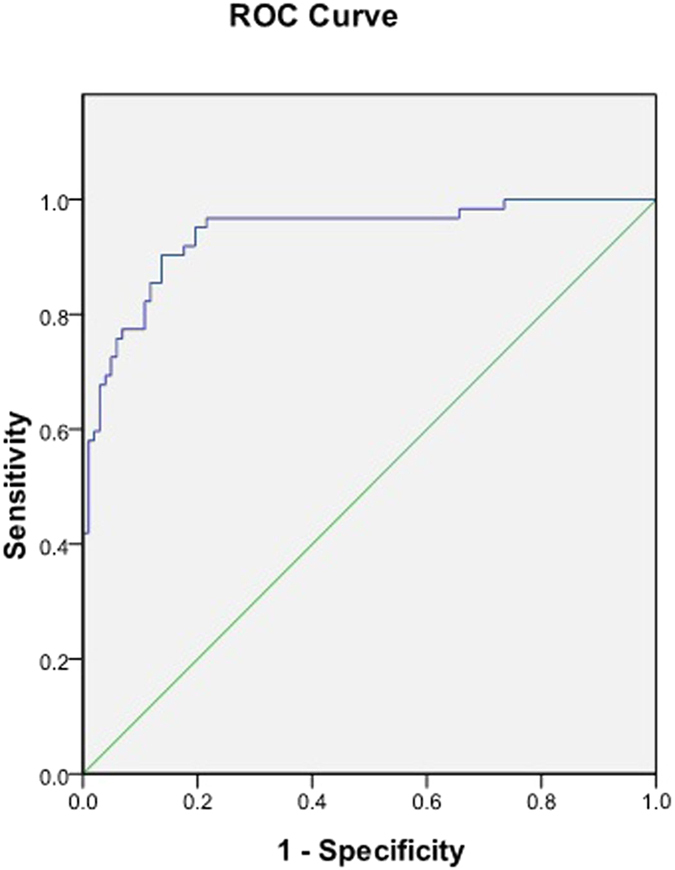
Combination of the ROC curve of three miRNAs for diagnosing IgAN. Normal control, n = 82; disease control, n = 40; IgAN group, n = 93.

ROC curve analysis in all included patients showed that these three urinary sediment miRNAs (miR-25-3p, miR-144-3p and miR-486-5p) had good specificity and sensitivity for the diagnosis of IgAN (Table 1), in which the AUC value of miR-486-5p was the largest, reaching 0.935 (Fig. 3). The combination of the three miRNAs could increase the AUC value to 0.940 for the diagnosis of IgAN (Fig. 4).

### Urinary sediment miRNAs for prognosis of IgAN

To validate the predictive value of urinary sediment miRNAs for the prognosis and outcome of IgAN, patients with IgAN were followed for an average period of 13.88 ± 6.00 months. Compared with baseline values, 24-hour urine protein values were significantly decreased, while no significant difference was found in eGFR levels (Supplementary Table S1). The miR-144-3p level was positively correlated with the change in eGFR (r = 0.286, P = 0.020) and negatively correlated with the change in 24-hour urine protein (r = −0.259, P = 0.019). Moreover, the miR-25-3p level was positively correlated with the change in eGFR (r = 0.257, P = 0.038). These results suggested that in IgAN patients, higher urinary sediment miR-144-3p expression levels were associated with decreased urine protein and renal function improvement. Higher levels of miR-25-3p expression were associated with renal function improvement. In the follow-up data of 85 patients in the IgAN group, 38.82% (n = 33) of these patients reached complete remission (CR) at the end of follow-up. However, none of urinary sediment miRNA levels differed significantly between the groups with and without CR (Supplementary Table S2).

### Urinary sediment miR-25-3p, miR-144-3p and miR-486-5p were mainly derived from erythrocytes

Using CD235a immunomagnetic technology to separate urinary erythrocytes, urinary sediment can be divided into total urinary sediment, a positive sorting group (urinary erythrocyte group) and a negative sorting group (group without erythrocytes). Using the comparative 2-ΔΔ CT (threshold cycle) method (threshold cycle values normalized by U6), 2-ΔΔ CT values of miR-25-3p (P < 0.01), miR-144-3p (P < 0.01) and miR-486-5p (P < 0.01) were significantly higher in the positive sorting group than in either the negative sorting group or the total urinary sediment group (Table 2). Moreover, the original CT values of these three miRNAs (all of P value < 0.001) were in the following order: negative sorting group >positive sorting group >total urinary sediment group. That meant that the descending order of absolute expression levels of these three miRNAs was total urinary sediment group >positive sorting group >negative sorting group. One possible reason is that the positive sorting would lose some red blood cells, and the absolute expression levels in positive sorting group were lower than those in total urinary sediment group (Table 2).

Mononuclear cells, human primary renal tubular epithelial cells and red blood cells were the main cell types in the urinary sediment. Our research also compared the baseline expression levels of miR-25-3p, miR-144-3p and miR-486-5p in these three cell types. The results showed that the original CT values of miR-25-3p (P < 0.001), miR-144-3p (P < 0.001) and miR-486-5p (P < 0.001) in red blood cells were significantly lower than in the other cell types (Table 3), which means that the baseline expression levels of these miRNAs in red blood cells were the highest.

### The increased expression of urinary erythrocyte miRNAs in IgAN patients was not associated with that in blood erythrocytes

To clarify whether the increase in urinary erythrocyte miRNAs in the IgAN group was due to the changes in blood erythrocytes, we compared the miRNA levels in blood erythrocytes between the IgAN and normal control groups. No significant differences were found in the original CT values of blood erythrocyte miR-144-3p (P = 0.075) and miR-486-5p (P = 0.090) between the two groups. The original CT values of blood erythrocyte miR-25-3p (P = 0.021) in the IgAN group were significantly higher than in the normal control group (Supplementary Table S3). Therefore, the higher expression of these miRNAs in the IgAN group was not due to the differences in blood erythrocytes between the IgAN and normal control groups.

To further clarify whether the increased miRNA expressions of urinary erythrocytes were influenced by kidney lesions or urinary microenvironmental changes in IgAN, blood erythrocytes from IgAN were added to their own urine to create a non-renal hematuria model of IgAN. The non-renal hematuria was comparable to the renal hematuria in the same IgAN patient with respect to similar erythrocyte numbers, incubation condition and incubation time. The major difference between the two models was that erythrocytes in renal hematuria were compressed out of IgAN kidney lesions. The results showed the levels of miR-25-3p, miR-144-3p and miR-486-5p of the erythrocytes in the renal hematuria group (P < 0.001) were significantly higher than in the non-renal hematuria model group (Supplementary Table S4).

### Comparison of urinary microvesicle miRNAs in the IgAN group and normal control group

We hypothesized that urinary erythrocyte miRNAs could have some biological effects on renal parenchymal cells by secreting miRNA-containing microvesicles. We examined the miRNAs of urinary supernatant microvesicles both in the IgAN and normal control groups. The results showed that the absolute expression levels of miR-144-3p (P = 0.042) and miR-486-5p (P < 0.001) of the urinary supernatant microvesicles in the IgAN group were significantly higher than those in the normal control group, whereas miR-25-3p (P = 0.192) showed no significant difference (Table 4). In addition, the original CT miR-486-5p value (28.954±0.925) of the urinary supernatant microvesicles in the IgAN group was less than 30, which reported a higher relative content (Table 4).

## Discussion

IgA nephropathy is the most common primary glomerulonephritis in the world^1^. The long-term prognosis of IgAN is not favorable; approximately 30–40% of IgAN patients progress into ESRD within 20 years^10^. The analysis of urinary sediment has some advantages, such as being non-invasive and easy to obtain frequently. Urinary sediment miRNAs may reflect the changes in renal tissue in IgAN. Therefore, they have the potential to be used as specimens to search for biomarkers of IgAN^11^. Compared with normal controls, 214 differentially expressed miRNAs in urinary sediment were found in IgAN by miRNA chip assay.

We selected and further analyzed three significantly elevated miRNAs in the IgAN group as candidate miRNA biomarkers. The biological functions of miR-25-3p^12^,^13^, miR-144-3p^14^ and miR-486-5p^15^,^16^ include promoting the antiapoptotic ability of cells, inhibiting PTEN activity and maintaining NF-κB activity. Another three that significantly decreased miRNAs, including miR-135a-3p^17^,^18^, miR-150-3p^19^ and miR-638^20^, in the IgAN group were also selected, which could promote cell apoptosis, promote renal fibrosis and inhibit PDGF-BB-induced cell proliferation. The qPCR results of the confirmation cohort were completely consistent with the trend in chip analysis. We then selected four miRNAs (miR-25-3p, miR-144-3p, miR-486-5p and miR-135a-3p), whose p-values were less than 0.001 and the fold changes more than 3 times, to be further validated in the expanded validation cohort. The results showed that urinary sediment miR-25-3p, miR-144-3p and miR-486-5p may be specific biomarkers for IgAN. ROC curve analysis showed that these three urinary sediment miRNAs had good specificity and sensitivity for the diagnosis of IgAN. The combination of the three miRNAs could increase the AUC value to 0.940 in the diagnosis of IgAN. Because of the relatively small sample size, further verifications are needed.

It would be of interest to study whether these urinary sediment miRNAs predict the prognosis and outcome of IgAN. The result showed the baseline level of miR-144-3p and miR-25-3p had significant positive correlations with the changes in eGFR. For the whole group of IgAN, eGFR levels had no significant difference during the follow up period, but the positive correlation indicated that the miR-144-3p and miR-25-3p levels may reflect the change trend of eGFR of each IgAN patient. That is, the miR-144-3p and miR-25-3p levels may also reflect the change trend of renal function improvement of each IgAN patient. The baseline level of miR-144-3p was negatively correlated with the 24-hour urine protein change, but we found the slightly decreased urine protein did not reach the defined of CR. At the end of the follow-up time, no significant differences in the urinary sediment miRNA levels at baseline were found between IgAN patients with and without clinical remission.

Previous studies showed that Fas as a direct target of miR-25-3p. Fas protein expression was downregulated by miR-25-3p overexpression^21^. And the over expression of miR-25-3p could alleviate aggregated IgA1 (AIgA1) induced apoptosis of podocytes in IgAN^22^. So the miR-25-3p may have some positive effect on improvement renal function. Research confirmed that the mammalian target of rapamycin (mTOR) was a direct target of miR-144-3p *in vitro*^23^. mTOR inhibition could prevent an additional increase in proteinuria and protect kidney function in a model of IgAN^24^. Further studies are needed to help uncover the mechanism underlying these relationships.

Recent research has indicated that red blood cells contain abundant miRNAs. Mature red blood cell miRNA accounted for the majority of the entire miRNA in the blood^8^,^9^. When we analyzed the correlation between miRNA expression levels and clinical parameters, we found the levels of miR-25-3p, miR-144-3p and miR-486-5p had significant differences between macroscopic hematuria group and microscopic hematuria group. So it reminded us to test whether these miRNAs were came from erythrocytes. CD235a is a surface biomarker of mature red blood cells. By using CD235a immunomagnetic technology, urinary sediment can be divided into the total urinary sediment, a positive sorting group and a negative sorting group. The positive sorting group had significantly higher levels of miR-25-3p, miR-144-3p and miR-486-5p than the negative sorting group. The positive sorting group accounted for the majority of the miRNA levels in the total urinary sediment. In addition, the baseline levels of miR-25-3p, miR-144-3p and miR-486-5p in red blood cells were the highest among the main cell types in the urinary sediment. Therefore, urinary sediment miR-25-3p, miR-144-3p and miR-486-5p are mainly derived from urinary erythrocytes.

We further compared the levels of these miRNAs in blood erythrocytes between the IgAN group and the normal control group. However, we found no differences in blood erythrocytes. Then, we created a model of non-renal hematuria in IgAN patients and found that the levels of miR-25-3p, miR-144-3p and miR-486-5p in the erythrocytes in the renal hematuria group were significantly higher than in the non-renal hematuria model group. Based on these results, neither the inter-group differences in blood erythrocytes nor the changes after the erythrocytes entered into the urine accounted for the differential expressions. We speculated that the increased miRNA levels of urinary erythrocytes might be associated with erythrocytes compressed out of renal lesions in IgAN.

It is still unknown whether the urinary erythrocyte miRNAs affect renal parenchymal cells and lead to pathological changes. Recent studies demonstrated that extracellular miRNA in microvesicles or exosomes may function in cell-to-cell communication^25^,^26^,^27^. A variety of cell types release microvesicles or exosomes, collectively termed extracellular vesicles^28^. The hematology study confirmed that red blood cells could secrete vesicles^29^. In normal circumstances, RBC-derived vesicles accounted for approximately 7.3% of the whole blood vesicles, while platelet-derived vesicles accounted for 38.5%. The endothelial-derived vesicles had the largest number, accounting for 43.5%^29^. However, under conditions of hemolysis, such as in sickle cell anemia^30^, thalassemia^31^,^32^, or malaria^33^, the numbers of RBC vesicles in circulation increase and could be predominant. We assumed that when urinary red blood cells are compressed out while exiting an impaired nephron due to mechanical damage or high osmotic pressure, this would result in the increased generation of RBC-derived vesicles and eventually lead to an increased expression of RBC-derived miRNAs in the urine supernatant. A previous study on the miRNA array results of IgAN renal tissue^34^ found that the miR-486-5p expression in IgAN renal tissue was 6.99 times higher than in the normal control group, which supports the hypothesis that blood erythrocytes secrete miRNA-containing vesicles when passing through impaired nephrons. These vesicles might be taken up by renal parenchymal cells and exert biological impacts affecting the occurrence or development of IgAN.

There were some limitations in this study. First, the sample size was relatively small. These results should be validated in large multicenter cohorts. Second, the impact of the urinary vesicles miR-144-3p and miR-486-5p on IgAN renal tissue and the possible mechanism should be investigated in future experiments.

In conclusion, this study showed that urinary sediment miR-25-3p, miR-144-3p and miR-486-5p could be non-invasive candidate biomarkers of IgA nephropathy. Urinary sediment miR-25-3p, miR-144-3p and miR-486-5p were mainly derived from urinary erythrocytes. The increased expression of urinary erythrocyte miRNAs was not associated with blood erythrocytes in IgAN, but they were associated with increased urinary supernatant microvesicles. The increased miRNAs of urinary erythrocytes might play biological roles in the development of IgAN.

## Methods

### Sample collection

A total of 93 patients with biopsy-proven IgAN were included, and 82 participants of the normal control group were matched by sex and age. In addition, 40 patients of the disease control group were also included: 15 patients with membranous nephropathy (MN), 8 patients with focal segmental glomerular sclerosis (FSGS), 5 patients with minimal change nephrosis (MCN), 5 patients with Henoch-Schonlein purpura nephritis (HSPN), and 2 patients with renal amyloidosis. The baseline characteristics of the patients enrolled in the study were shown in Supplementary Table S5. The study was carried out according to the principles of the Declaration of Helsinki and was approved by the Ethics Committee of the Chinese PLA General Hospital. Informed consent was obtained from all patients before entry into the study. Patients were followed up every three months. Data were recorded during follow-up, including 24-hour proteinuria, urinary red blood cells, serum creatinine, and eGFR. eGFR was estimated with the Asian modified CKD-EPI equation^35^. Proteinuria remission: the mean 24-hour proteinuria was less than 0.3 g/d. Hematuria remission: the mean count of urinary sediment red blood cells was less than 3/high-power field. Complete remission (CR) was defined as negative urine protein and urinary red blood cells in three consecutive examinations.

### Urine collection and RNA extraction

Whole stream early morning urine specimens collected from patients and controls were processed within 4 hours after collection at 4 °C. Each urine sample was centrifuged at 3000 g for 30 minutes and 13000 g for 15 minutes at 4 °C^4^. The urine supernatant was stored at −80 °C. TRIzol (Vitrogen, USA) was used for the extraction of total RNA from urinary sediment according to the manufacturer’s protocol.

### miRNA microarray and quantitative real-time PCR

We performed miRNA microarray analysis in nine IgAN patients (Lee’s grade I-II: 3, grade III: 3, grade IV-V: 3) and three healthy participants using the Agilent Human miRNA Microarrays V19.0 according to the manufacturer’s protocol.

RNA concentration and purity were determined using the NanoDrop 2000c spectrophotometer (Thermo Fisher Scientific, Waltham, MA, USA). We used the miRcute miRNA First-Strand cDNA Synthesis Kit (Tiangen Biotech, Beijing, China) and miRcute miRNA qPCR Detection kit (Tiangen Biotech, Beijing, China) for reverse transcription and quantitative detection. Urinary sediment hsa-U6, hsa-miR-25-3p, hsa-miR-144-3p, hsa-miR-486-5p, hsa-miR-135a-3p, hsa-miR-150-3p and hsa-miR-638 were quantified by real-time quantitative polymerase chain reaction (RT-QPCR) using the ABI Prism 7500 Sequence Detection System (Applied Biosystems, Foster City, CA, USA). All reactions were run in triplicate. Small RNA has-U6 (Applied Biosystems) was used as a house-keeping gene to normalize the microRNA expression^36^, and miRNA abundance was presented as the threshold cycle (Ct) values normalized to U6. The ΔΔCT method for relative quantitation was used to calculate the differences in the expression level for each target among the samples.

### Separation and purification of urinary erythrocytes

The urinary sediment was added to 1 ml PBS solution at 4 °C and mixed by pipetting. Each type of cell in the urinary sediment was counted by Urinary Sediment Quantitative Analyxer (SanHo Vivien Medical Products Co., Tianjin, China), and the total cell number and the number of urinary erythrocytes were recorded. The urinary sediment was divided into two parts: one for the total urinary sediment, another separation for urinary erythrocytes. CD235a (the biomarker for mature human RBC) magnetic beads (Miltenyi Biotec, CA) were used to divide the urinary sediment into a positive sorting group (with urinary erythrocytes) and a negative sorting group (without erythrocytes). We checked again whether the positive sorting group contained karyocytes or the negative sorting group contained RBCs to ensure the purity of separation and purification.

### Separation and purification of blood erythrocytes

Blood was obtained by venipuncture and collected into EDTA-containing tubes. After centrifugation, the buffy coat was carefully removed from the whole blood. One ml of the under layer of erythrocytes was siphoned and mixed at 1:1 with the buffer (Hanks’ balanced salt solution with 2% FCS and 0.02% NaN3), then carefully added to 1 ml Ficoll-Hypaque (Ficoll-Paque Plus, GE Healthcare) and centrifuged at 1500 rpm (with a radius of 15 cm horizontal rotor) for 20 minutes. The mixture from top to bottom was divided into four layers. The fourth layer was the red blood cell layer. The fourth (red blood cell) layer was aspirated and the above steps were repeated three times.

### Preparation of non-renal hematuria model

A 50 ml morning urine specimen and 3 ml of EDTA-containing blood was collected from each patient. The blood erythrocytes were separated and purified using the above-mentioned method. The morning urine specimen was centrifuged to remove the urinary sediment. Blood erythrocytes were added to the urinary supernatant, and constituted up to approximately 30 red blood cells per high-power field. The sample was incubated at 37 °C for two hours.

### Separation and purification of peripheral blood mononuclear cells (PBMCs) and human primary renal tubular epithelial cells

PBMCs were isolated by density separation over a Ficoll-Hypaque (Ficoll-Paque Plus, GE Healthcare) gradient (460 g for 30 minutes) as previously described^37^. Macroscopically and histologically normal kidney tissues were obtained aseptically from adult human kidneys that were removed surgically because of small (<6 cm) renal adenocarcinomas, pelviureteric transitional cell carcinoma or benign angiomyolipoma. All experiments were carried out strictly in accordance with international ethical guidelines. The use of human renal tissue had been approved by the Human Medical Research Ethics Committee at the Chinese PLA General Hospital and Medical School of the Chinese PLA. Informed consent was obtained from patients prior to their surgical procedures. Extraction of human primary renal tubular epithelial cells was performed as described^38^.

### Extraction of urinary supernatant vesicles

Urine samples were centrifuged at 2000 g for 20 minutes and at 13,500 g for 20 minutes at 4 °C, and the urinary sediment discarded. The urinary supernatant continued to be centrifuged at 150,000 g for 60 minutes at 4 °C^39^.

### Statistical analyses

The SPSS 17.0 and Graphpad Prism 5.01 for Windows (Graphpad Software Inc., San Diego, CA, USA) were used for the statistical analysis and graphing. All miRNA values were expressed as the mean ± standard deviation (SD) of the data obtained from at least three independent experiments. Each dataset was analysed for normality using a Shapiro-Wilk test. Two-tail student’s t-test was used to compare miRNA levels between two groups where appropriate. Nonparametric Mann–Whitney or Kruskal–Wallis rank tests were used for testing the parameters of those data that were not normally distributed. We used one-way analysis of variance (ANOVA) followed by the Student-Newman-Keuls posttest to check if a significant difference in miRNA levels existed between multiple groups. Receiver operating characteristic (ROC) curves and the area under the ROC curve (AUC) were calculated to evaluate the sensitivity and specificity of the data for diagnosing IgA nephropathy. The combined diagnostic capabilities of multiple miRNAs were calculated using ROC curve analysis as previously described^40^. The results were considered statistically significant at P < 0.05. All probabilities were two-tailed.

## Additional Information

**How to cite this article**: Duan, Z.Y. *et al*. Selection of urinary sediment miRNAs as specific biomarkers of IgA nephropathy. *Sci. Rep.* **6**, 23498; doi: 10.1038/srep23498 (2016).

## Supplementary Material

Supplementary Information

## Figures and Tables

**Table 1:** Features of the receiver operating characteristic curve of miRNAs for diagnosing IgAN.

	**AUC**	**95% CI**	**P Value**	**Optimal cut-off value**	**Sensitivity (%)**	**specificity (%)**
miR-25-3p	0.884	0.831–0.937	<0.001	2-ΔΔct = 0.045	87.1	79.4
miR-144-3p	0.869	0.811–0.927	<0.001	2-ΔΔct = 0.006	82.3	77.5
miR-486-5p	0.935	0.897–0.973	<0.001	2-ΔΔct = 0.035	93.5	85.3

AUC, the area under the ROC curve; CI, confidence interval. Normal control, n = 82; disease control, n = 40; IgAN group, n = 93.

**Table 2:** Comparison of miRNA levels between the total urinary sediment group, positive sorting group and negative sorting group.

**microRNAs**	**Positive sorting group**	**Negative sorting group**	**Total urinary sediment group**	**P Value**
Normalized by U6				
miR-25-3p	1.3277±1.1585	0.0053±0.0047	0.2466±0.2873	0.002
miR-144-3p	1.0497±0.9299	0.0010±0.0013	0.0890±0.0764	0.001
miR-486-5p	1.6787±1.3294	0.0064±0.0105	0.1971±0.1951	0.001
The original CT values				
miR-25-3p	26.105±0.927	31.173±1.066	24.500±1.381	<0.001
miR-144-3p	26.675±1.327	33.967±1.156	26.071±1.408	<0.001
miR-486-5p	25.826±1.209	31.903 ± 2.166	24.632±1.26	<0.001

Positive sorting group, n = 11; negative sorting group, n = 11; total urinary sediment group, n = 11.

**Table 3:** Comparison of miRNA expression levels between mononuclear cells, human primary renal tubular epithelial cells and red blood cells.

**microRNAs**	**Red blood cells**	**Mononuclear cells**	**Human primary renal tubular epithelial cells**	**P Value**
The original CT values				
miR-25-3p	16.862±2.164	27.095±0.266	24.65±1.655	<0.001
miR-144-3p	14.098±1.939	31.835±0.284	33.975 ± 1.160	<0.001
miR-486-5p	16.834±2.791	32.445±0.962	30.039±0.774	<0.001

Red blood cell control, n = 10; mononuclear cell, n = 8; human primary renal tubular epithelial cell group, n = 10.

**Table 4:** Comparison of the miRNA levels of urinary supernatant microvesicles between the IgAN group and normal control group.

**microRNAs**	**IgAN group**	**Normal control group**	**P Value**
the original CT values			
miR-25-3p	31.493±1.958	32.91±0.407	0.192
miR-144-3p	35.554±1.429	37.306±0.478	0.042
miR-486-5p	28.954±0.925	34.067±1.426	<0.001

Normal control, n = 7; IgAN group, n = 10.
