# Supplementary material for: Selection of urinary sediment miRNAs as specific biomarkers of IgA nephropathy
Source: Sci Rep. 2016 Mar 22;6:23498. doi: 10.1038/srep23498 (PMC4802218; doi:10.1038/srep23498)
Supplement: Supplementary Information [file srep23498-s1.pdf]

# Selection of urinary sediment miRNAs as specific biomarkers of IgA nephropathy

ZhiYu Duan[1], Guangyan Cai[1]*, Ru Bu[1], Yang Lu[1], Kai Hou[1] and Xiang-Mei Chen[1].

**Affiliation:** [1]Department of Nephrology, Chinese PLA General Hospital, State Key Laboratory of Kidney Diseases, National Clinical Research Center for Kidney Diseases , Beijing , PR China.

**Supplementary Figure S1**

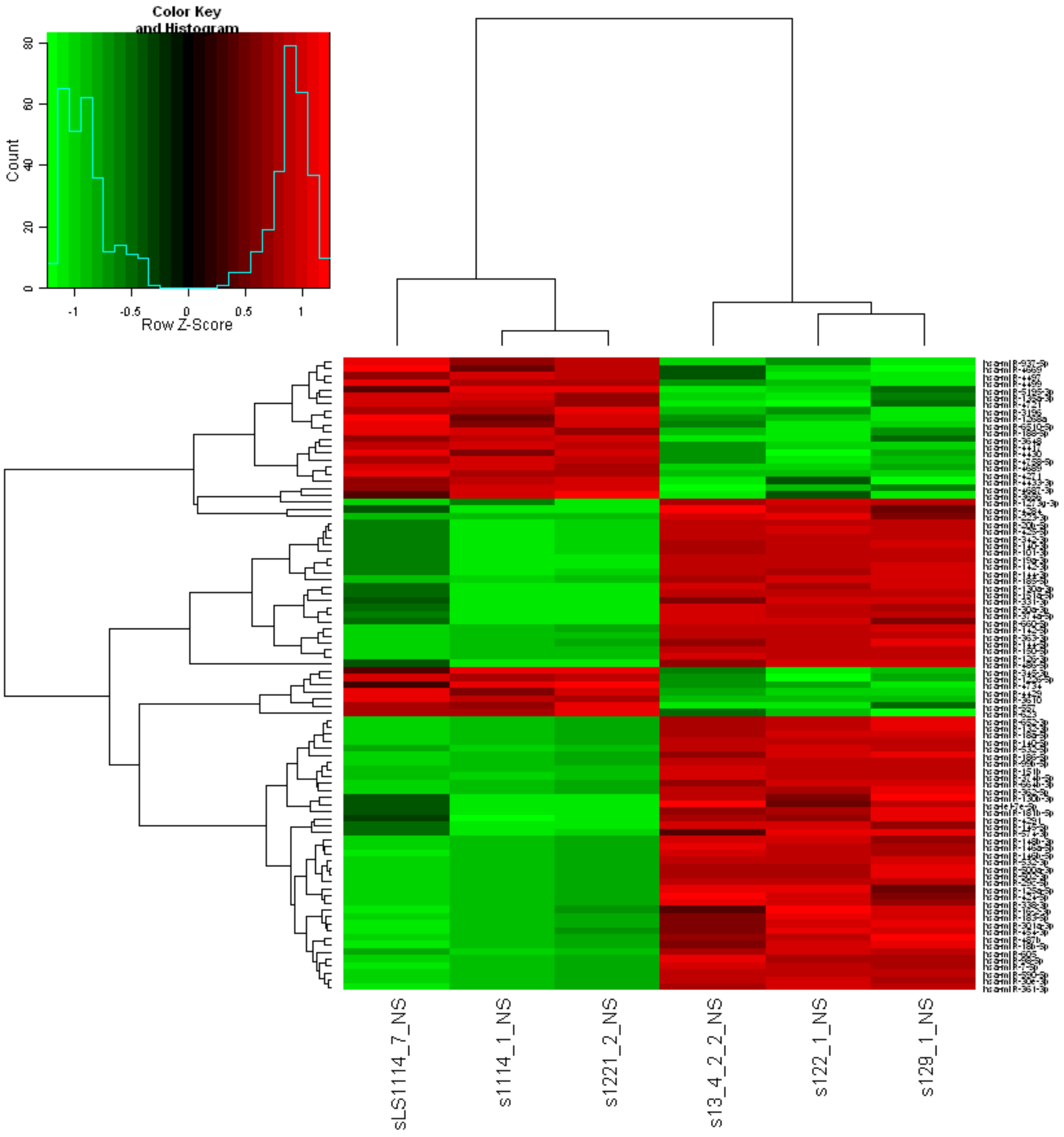


**Supplementary Figure S1-1. Comparison of urinary sediment miRNAs expression profiles between normal control group (left) and the IgAN LEE I-II group (right) (a list of p <0.01 indicators).** Normal control, n=3; IgAN LEE I-II group, n=3.

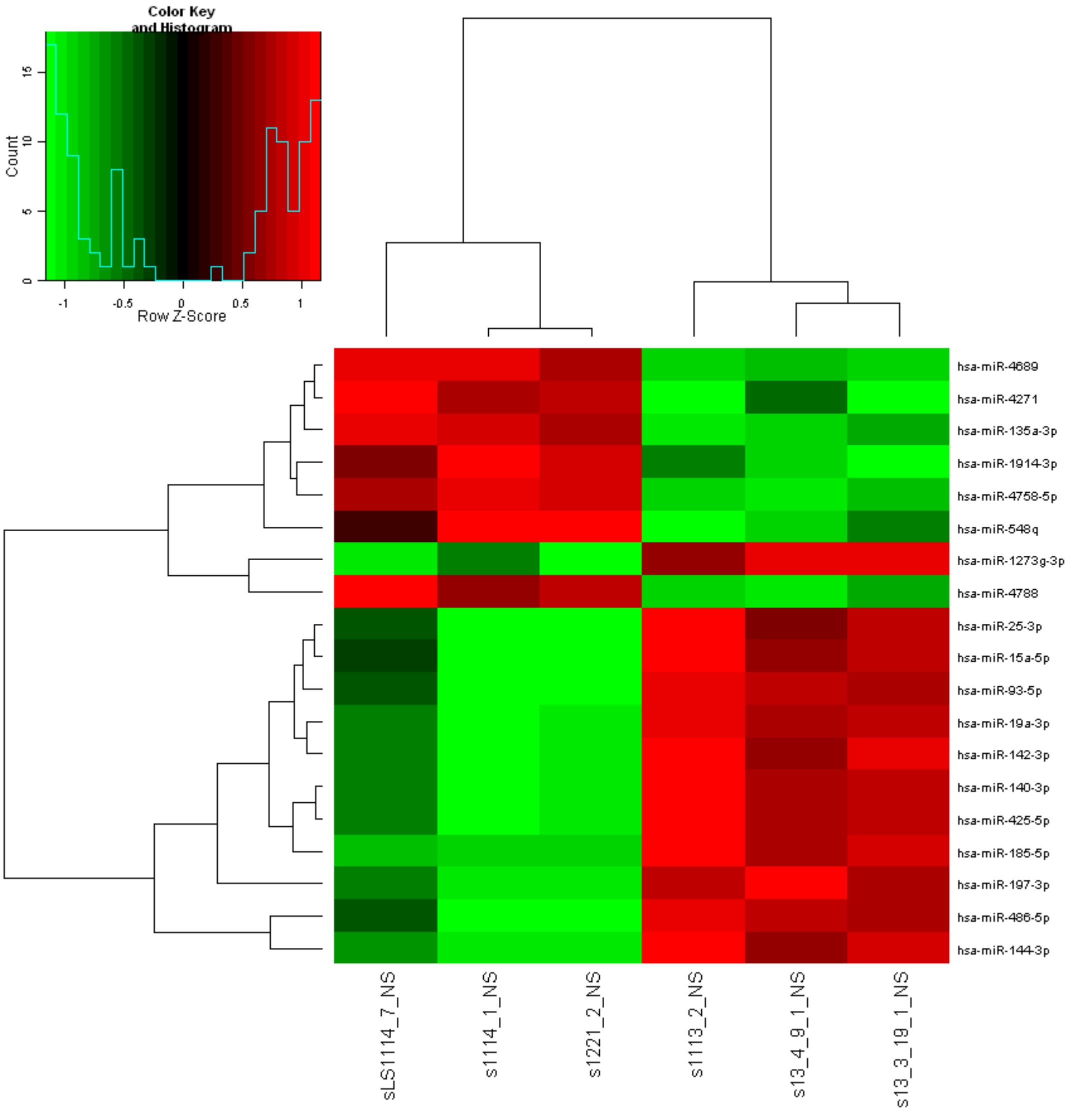


**Supplementary Figure S1-2. Comparison of urinary sediment miRNAs expression profiles between normal control group (left) and the IgAN LEE III group (right) (a list of p <0.01 indicators).** Normal control, n=3; IgAN LEE III group, n=3.

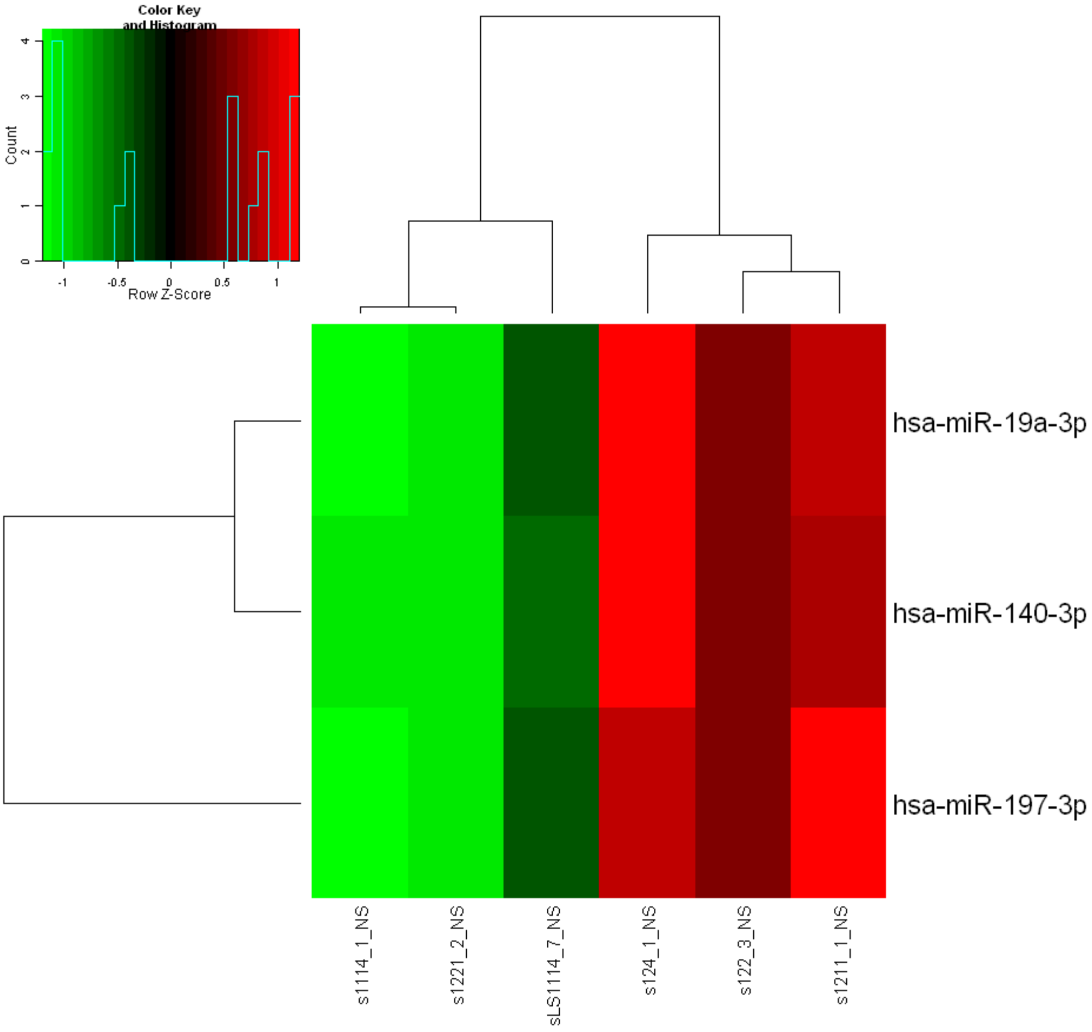


**Supplementary Figure S1-3. Comparison of urinary sediment miRNAs expression profiles between normal control group (left) and the IgAN LEE IV-V group (right) (a list of p <0.01 indicators).** Normal control, n=3; IgAN LEE IV-V group, n=3.

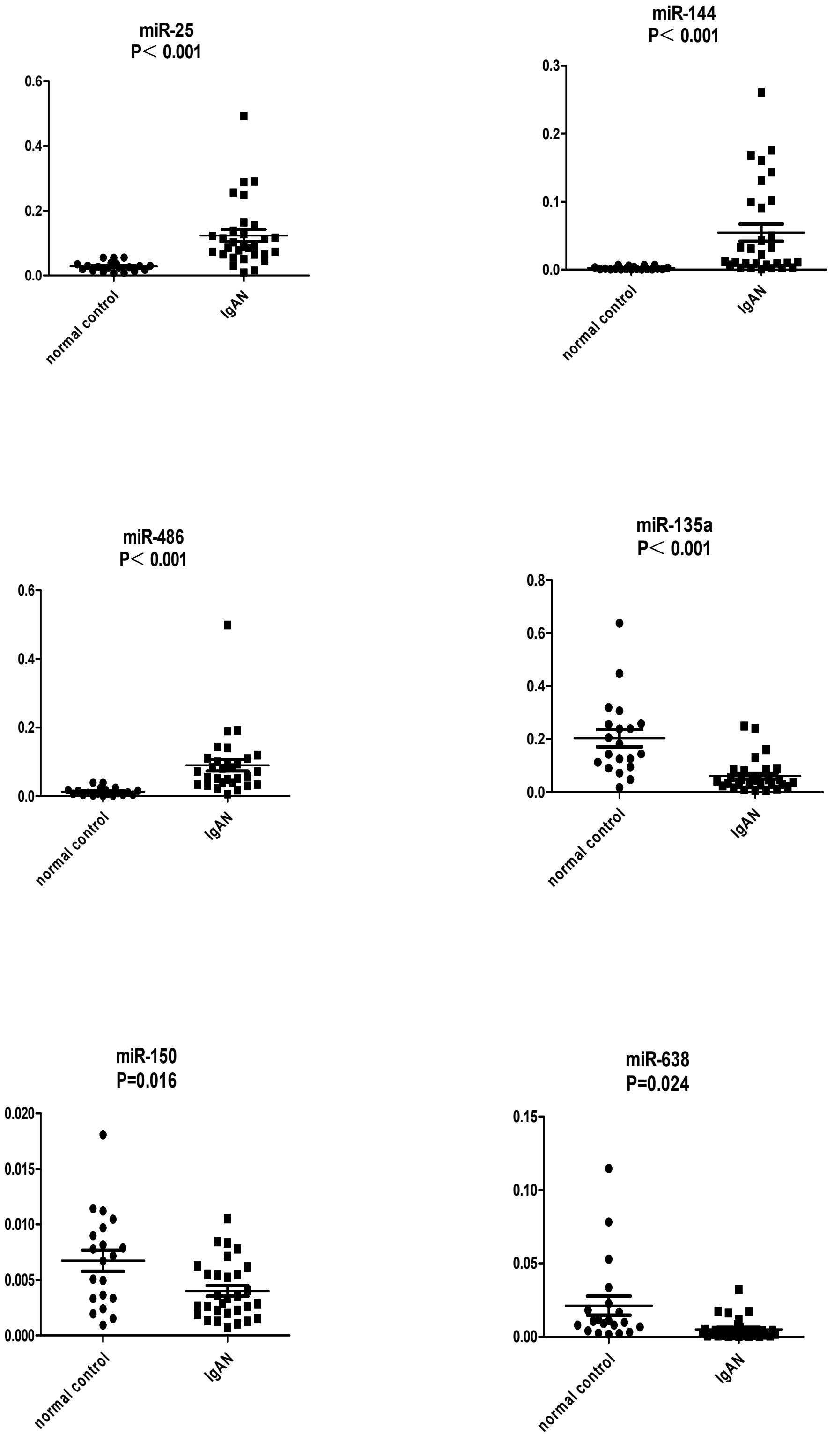
miR-25
P< 0.001
0.6
0.4
0.2
0.0
normal control
IgAN
miR-144
P< 0.001
0.3
0.2
0.1
0.0
normal control
IgAN
miR-486
P< 0.001
0.6
0.4
0.2
0.0
normal control
IgAN
miR-135a
P< 0.001
0.8
0.6
0.4
0.2
0.0
normal control
IgAN
miR-150
P=0.016
0.020
0.015
0.010
0.005
0.000
normal control
IgAN
miR-638
P=0.024
0.15
0.10
0.05
0.00
normal control
IgAN

**Supplementary Figure S2. Comparison of urinary sediment miRNA expression levels between the IgAN group and normal control group in a confirmation cohort.** Normal control, n=20; IgAN group, n=30. The P values were calculated by 2-tailed Student t test. N: number.

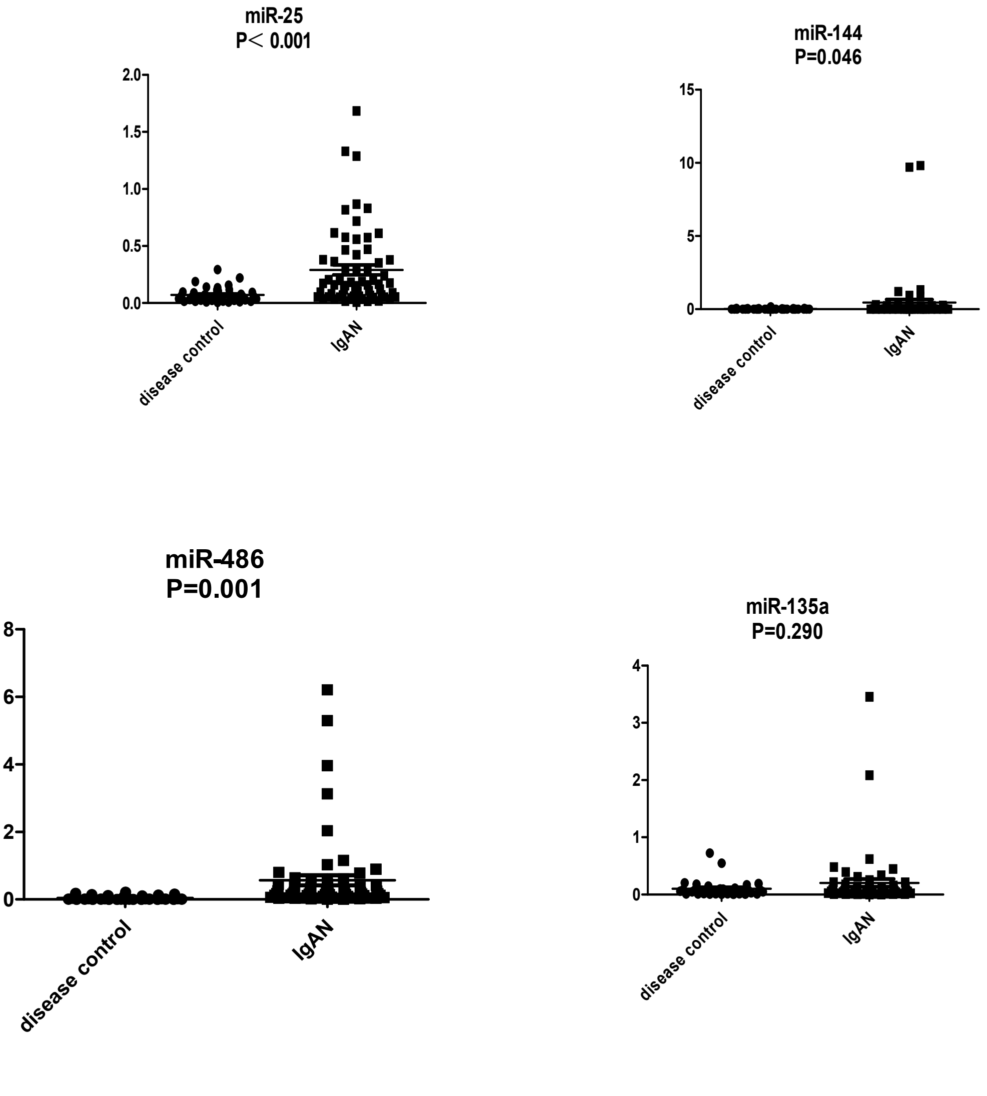

**Supplementary Figure S3. Comparison of urinary sediment miRNA expression levels between the IgAN group and disease control group.** Disease control, n=40; IgAN group, n=63. The P values were calculated by 2-tailed Student t test. N: number.

**Supplementary Table S1. 24-hour proteinuria, serum creatinine and eGFR at each point of follow-up time.**

| | 24-hour proteinuria | eGFR |
|---|---|---|
| 3 months follow-up | 1.20±1.46 | 92.41±37.49 |
| vs baseline value | P=0.144 | P=0.482 |
| 6 months follow-up | 0.77±0.98 | 92.34±35.62 |
| vs baseline value | P＜0.001 | P=0.504 |
| 12 months follow-up | 0.91±1.66 | 93.17±33.02 |
| vs baseline value | P=0.045 | P=0.475 |
| The end of follow-up | 0.65±0.87 | 96.60±42.14 |
| vs baseline value | P＜0.001 | P=0.193 |

eGFR, estimated glomerular filtration rate calculated with the Chronic Kidney Disease Epidemiology Collaboration (CKD-EPI) equation (ml/min per 1.73m2). The follow-up patients of IgAN group, n=85.

**Supplementary Table S2. Comparison of urinary sediment miRNA values between the CR group and the non-CR group at the end of follow-up.**

| Normalized by U6 | The CR group | The non-CR group | P value |
|---|---|---|---|
| miR-25-3p | 0.2257±0.2833 | 0.1940±0.2028 | P=0.551 |
| miR-144-3p | 0.0936±0.1700 | 0.1052±0.2189 | P=0.798 |
| miR-486-5p | 0.3925±0.8429 | 0.1623±0.1988 | P=0.132 |

CR, complete remission. CR control, n=33; non-CR group, n=52.

**Supplementary Table S3. Comparison of blood erythrocyte miRNAs between the IgAN group and normal control group.**

| microRNAs | IgAN group | Normal control group | P Value |
|---|---|---|---|
| The original CT values | | | |
| miR-25-3p | 18.218±1.942 | 15.506±1.457 | 0.021 |
| miR-144-3p | 15.085±1.911 | 13.110±1.510 | 0.075 |
| miR-486-5p | 18.197±2.552 | 15.470±2.483 | 0.090 |

Normal control, n=8; IgAN group, n=8.

**Supplementary Table S4. Comparison of urinary erythrocyte miRNAs between the IgAN renal hematuria group and the IgAN non-renal hematuria model group.**

| microRNAs | IgAN renal hematuria group | Non-renal hematuria model group | P Value |
|---|---|---|---|
| The original CT values | | | |
| miR-25-3p | 26.105±0.927 | 34.165±2.108 | ＜0.001 |
| miR-144-3p | 26.675±1.327 | 33.708±2.268 | ＜0.001 |
| miR-486-5p | 25.826±1.209 | 32.663±1.839 | ＜0.001 |

IgAN renal hematuria group, n=11; non-renal hematuria model group, n=11.

**Supplementary Table S5. The baseline characteristics of IgAN patients and control group.**

| | Confirmation cohort | | Validation cohort | | |
|---|---|---|---|---|---|
| | IgAN patients | Normal control | IgAN patients | Normal control | Disease control |
| Participants | 30 | 20 | 63 | 62 | 40 |
| Male/Female | 18/12 | 10/10 | 33/30 | 34/28 | 16/24 |
| Age(yr) | 34.37±10.334 | 33.45±7.28 | 34.92±10.338 | 34.21±7.41 | 43.07±17.457 |
| Serum Creatinine(umol/L) | 104.84±47.67 | ND | 106.31±56.34 | ND | 78.55±19.55 |
| Estimate GFR(ml/min/1.73m$^2$) | 91.04±37.39 | ND | 88.57±34.67 | ND | 99.44±26.67 |
| Proteinuria(g/24h) | 1.95±1.81 | 0.11±0.14 | 1.32±1.27 | 0.09±0.16 | 4.06±2.8 |
| Hematuria(Y/N) | 21/9 | 0/20 | 47/16 | 0/62 | 24/16 |

Unless otherwise noted, values are expressed as mean±SD. eGFR, estimated glomerular filtration rate calculated with the Chronic Kidney Disease Epidemiology Collaboration (CKD-EPI) equation (ml/min per 1.73m2); IgAN, IgA nephropathy; ND, not determined.
